# Characterization of the *sdw1* semi-dwarf gene in barley

**DOI:** 10.1186/s12870-016-0964-4

**Published:** 2017-01-13

**Authors:** Yanhao Xu, Qiaojun Jia, Gaofeng Zhou, Xiao-Qi Zhang, Tefera Angessa, Sue Broughton, George Yan, Wenying Zhang, Chengdao Li

**Affiliations:** 1 Hubei Collaborative Innovation Center for Grain Industry/College of Agriculture, Yangtze University, Jingzhou, Hubei 434000 China; 2 Western Barley Genetics Alliance, Murdoch University, Murdoch, WA6150 Australia; 3 Key Laboratory of Plant Secondary Metabolism and Regulation of Zhejiang Province /College of Life Sciences, Zhejiang Sci-Tech University, Hangzhou, 310018 China; 4 Department of Agriculture and Food Government of Western Australia, South Perth, WA6150 Australia; 5 College of Horticultural and Forestry Sciences, Huazhong Agricultural University, Wuhan, 430070 China

**Keywords:** *sdw1*, Functional gene, Allelic variation, Diagnostic marker, Functional compensation

## Abstract

**Background:**

The dwarfing gene *sdw1* has been widely used throughout the world to develop commercial barley varieties. There are at least four different alleles at the *sdw1* locus.

**Results:**

Mutations in the gibberellin 20-oxidase gene (*HvGA20ox2*) resulted in multiple alleles at the *sdw1* locus. The *sdw1.d* allele from Diamant is due to a 7-bp deletion in exon 1, while the *sdw1.c* allele from Abed Denso has 1-bp deletion and a 4-bp insertion in the 5’ untranslated region. The *sdw1.a* allele from Jotun resulted from a total deletion of the *HvGA20ox2* gene. The structural changes result in lower gene expression in *sdw1.d* and lack of expression in *sdw1.a*. There are three *HvGA20ox* genes in the barley genome. The partial or total loss of function of the *HvGA20ox2* gene could be compensated by enhanced expression of its homolog *HvGA20ox1*and *HvGA20ox3*. A diagnostic molecular marker was developed to differentiate between the wild-type, *sdw1.d* and *sdw1.a* alleles and another molecular marker for differentiation of *sdw1.c* and *sdw1.a*. The markers were further tested in 197 barley varieties, out of which 28 had the *sdw1.d* allele and two varieties the *sdw1.a* allele. To date, the *sdw1.d* and *sdw1.a* alleles have only been detected in the modern barley varieties and lines.

**Conclusions:**

The results provided further proof that the gibberellin 20-oxidase gene (*HvGA20ox2*) is the functional gene of the barley *sdw1* mutants. Different deletions resulted in different functional alleles for different breeding purposes. Truncated protein could maintain partial function. Partial or total loss of function of the *HvGA20ox2* gene could be compensated by enhanced expression of its homolog *HvGA20ox1* and *HvGA20ox3.*

**Electronic supplementary material:**

The online version of this article (doi:10.1186/s12870-016-0964-4) contains supplementary material, which is available to authorized users.

## Background

Semi-dwarfism is a valuable and widely used trait in intensive agriculture. The high yield potential of semi-dwarf cultivars is attributed to their improved harvest index, lodging resistance, and more efficient utilization of the environment [1]. The green revolution, led by semi-dwarf varieties in wheat, was due to the introduction of the *Rht* gene, which encodes a mutant form of a DELLA protein, a gibberellin signaling repressor [2]. The green revolution in rice was due to semi-dwarf varieties carrying *sd1*, a single locus encoding a defective *gibberellin 20-oxidase-2* (*GA20ox2*) [3].

Semi-dwarf barley cultivars have been successfully used around the world. In China, more than 350 dwarf and semi-dwarf cultivars and entries have been developed since 1950, with an average 4.7-fold yield increase over landraces and older cultivars [4]. There are more than 30 types of dwarfs or semi-dwarfs described in barley, among which *semi-brachytic 1* (*uzu1*), breviaristatum-e (*ari-e*), and *semi-dwarf 1* (*sdw1*) are widely used in modern barley improvement [5, 6]. The *ari-e* mutant from Golden Promise has been used in several European cultivars and is located on chromosome 5HL [7]. The *uzu* gene is located on chromosome 3HL, which has been the major dwarfing gene used in East Asia barley breeding programs [8, 9]. The dwarfism controlled by *uzu* is caused by a missense mutation of a single nucleotide substitution in the *HvBRI1* gene, which reduces the response to brassinolide [9].

The *sdw1* locus has been widely used to develop modern barley varieties in Europe, North America, South America, and Australia. There are at least four alleles at the *sdw1* locus, which arose from separate mutation events: *sdw1.a* (originally named *sdw1*), *sdw1.c* (originally named *denso*), *sdw1.d* (Diamant) and *sdw1.e* (mutant line ‘Ris∅ no. 9265’) [10]. The *sdw1.c* allele was the first reported allele at the *sdw1* locus, a spontaneous mutant selected from barley cultivar Abed Denso [11]. The *sdw1.c* allele was successfully transferred to cultivars Deba Abed and Maris Mink, and later introduced into numerous barley crosses in Southern Swedish and Danish breeding programs [6]. The *sdw1.a* allele was induced by X-ray mutagenesis in a Norwegian six-rowed barley Jotun and has been used in Western USA, Canada, and Australia to breed semi-dwarf feed barley cultivars like Yerong and UC828 [12–14]. The *sdw1.d* allele, probably the most important for breeding, originated from a mutant selected in the M2 generation of cv. Valticky after X-ray treatment [6, 10, 11, 15]. The mutant was officially released in Czechoslovakia in 1965 as cv. Diamant, and this allele has been used for the successful release of more than 150 new malting barley cultivars in Europe [6, 15]. The *sdw1.d* allele has gained great acceptance in malting barley breeding programs in Europe, Canada, USA, and Australia, while the *sdw1.a* allele has been limited to feed barley varieties [14]. The fourth allele, *sdw1.e* (mutant line ‘Ris∅ no. 9265’) was found in the M2 generation of cv. Bomi after treatment with partially moderated fission neutrons in a reactor [10]. However, there are no reports of the use of this allele in variety development [6].

The *sdw1* locus is located on chromosome 3HL, but more distal from the centromere than *uzu1* [16]. Comparative genomic analysis revealed that the *sdw1* gene in barley is located in the syntenic region of the rice green revolution semi-dwarf gene *sd1*, encoding a gibberellin 20-oxidase enzyme [13]. However, it is not clear what the gene structure changes resulted in different functional alleles. The objectives of this study were to (i) confirm gibberellin 20-oxidase as the functional gene, (ii) provide a detailed molecular characterization of different alleles at the *sdw1* locus, (iii) understand how gene expression at the locus is regulated, and (iv) develop an allele-specific diagnostic marker for barley breeding programs.

## Results

### Cloning the *HvGA20ox2* gene from barley genomic DNA

A fragment of 4831 bp was isolated from the tall barley varieties AC Metcalfe, Hamelin, and Valticky following PCR amplification of genomic DNA (Additional file 1: Figure S1). Based on FGENESH gene annotation, the barley *HvGA20ox2* gene (3486 bp) contains three exons and two introns, with 1030 bp for exon 1, 325 bp for exon 2, 490 bp for exon 3, 173 bp for intron 1, and 1468 bp for intron 2. The coding sequence is 1242 bp in length, with a 371 bp 5’ untranslated region in exon 1 and a 232 bp 3’ untranslated region in exon 3 (Additional file 1: Figure S1). In addition, the isolated 4831 bp barley DNA fragment contains a 974-bp 5' upstream sequence and a 371-bp 3' downstream sequence of the *HvGA20x2* gene.

**Fig. 1:**
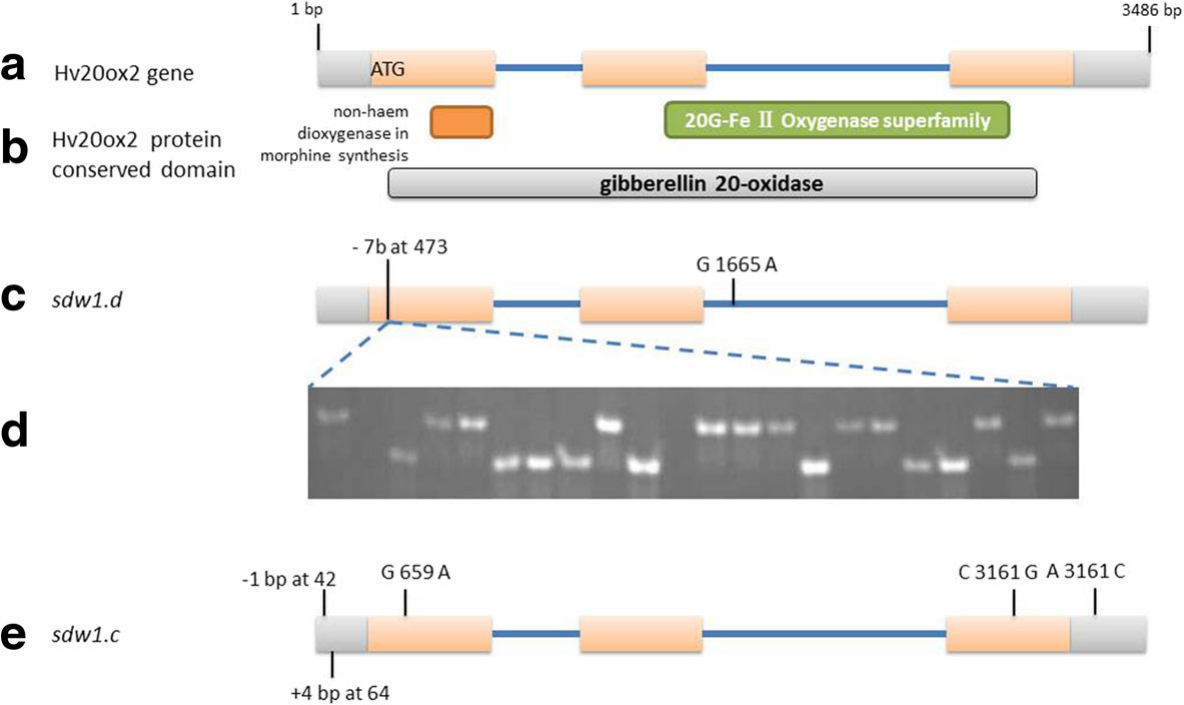
Allelic variations of *HvGA20ox2* gene in barley. **a**: structure of *HvGA20ox2* gene; **b**: conserved domain of *HvGA20ox2* protein; **c**: *sdw1.d* allele; **d**: verification of deletion in *sdw1.d* allele in a DH pupation of Baudin/AC Metcalfe; **e**: *sdw1.c* allele mutation

The putative protein of the *HvGA20ox2* gene has 414 amino acids. The predicted protein contains a conserved domain of the 2OG-Fe(II) oxygenase superfamily, non-haem dioxygenase in morphine synthesis, and gibberellin 20-oxidase (Fig. 1a, b).

The barley *HvGA20ox2* orthologous genes were identified by BLASTP in rice (*sd1 OsGA20ox2*, AAL87949), wheat (CDM85079.1), *Aegilops* (EMT17460), *Brachypodium* (XP003567337), maize (XP008654721), sorghum (XP002456751), *Setaria italica* (XP004970813) and *Arabidopsis* (*GA20ox1* gene, NP194272). The amino acid sequence identity of the predicted *HvGA20ox2* proteins in other grass species and *Arabidopsis* is listed in Additional file 2: Table S1. The predicted protein of the barley *HvGA20ox2* gene was more similar to wheat and *Aegilops* (94.0 and 95.4% identity, respectively) than maize and *Brachypodium* (74.4 and 74.7% identity, respectively). As expected, the lowest level of identity was found for *Arabidopsis* (46.9%).

**Fig. 2:**
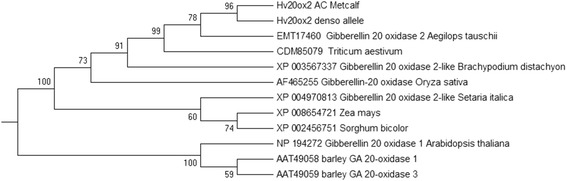
Phylogenetic trees of the predicted proteins of *HvGA20ox2* gene including the ortholog proteins

The barley *HvGA20ox1* (AAT49058) and *HvGA20ox3* (AAT49059) genes, previously isolated, are also involved in GA (gibberellic acid) biosynthesis [17]. The predicted protein of *HvGA20ox2* only shares 50.6 and 48.5% of sequence identity with *HvGA20ox1* (AAT49058) and *HvGA20ox3* (AAT49059), respectively. Phylogenetic trees of the predicted proteins of barley *HvGA20ox2* and the orthologous proteins *HvGA20ox1* and *HvGA20ox3* were constructed (Fig. 2).

### Allelic variation of *HvGA20ox2* in semi-dwarf barley

The nucleotide sequences of the *HvGA20ox2* gene from the three tall barley varieties (AC Metcalfe, Hamelin and Valticky) were identical. DNA sequences of the *HvGA20ox2* gene were isolated from Baudin and Diamant, two semi-dwarf barley varieties known to have the *sdw1.d* allele. No nucleotide differences were detected between Baudin and Diamant. A comparison between the three tall barley varieties and *sdw1.d* allele semi-dwarf barley (Baudin and Diamant) identified a 7-bp (GACTCCC) deletion in the coding region of exon 1, from position 473 to 479, in the *sdw1.d* allele (Fig. 1c). In addition, the previously detected A/G substitution was also confirmed in this study [13]. The deletion in the *sdw1.d* allele was predicted to cause coding frame shifts and premature translation termination. Sequence analysis showed that there are ten internal ‘ATG’ start sites in the *sdw1.d* coding sequence. Among them, three ‘ATG’ sites located in position 1026–1028 (exon 1),1232–1234 (exon 2) and 1334–1336 (exon 2) could translate to a truncated protein with a conserved domain of the 2-oxoglutarate (2OG) and Fe(II)-dependent oxygenase superfamily (Fig 1).

**Fig. 3:**
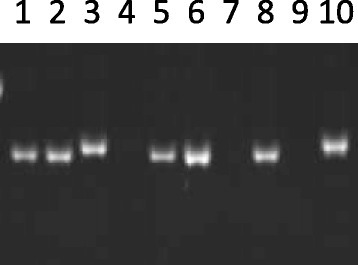
The 4-bp insertion in the *sdw1.c* allele amplified by the marker MC40861P in *HvGA20ox2* gene. Lanes 4, 7 and 9 represent the sdw1.a allele. DNA templates (from left to right): 1. AC Metcalfe, 2. Baudin, 3. Deba Abed, 4. Jotun, 5. Hamelin, 6. Triumph, 7. Yerong, 8. Diamont, 9. Jotun, 10. Maris Mink

Another important semi-dwarf allele of the *HvGA20ox2* gene is *sdw1.c* (originally named *denso*). The DNA sequence of *HvGA20ox2* was determined from a semi-dwarf barley Deba Abed. This allele did not have the *sdw1.d* (Diamant, also called as *denso* in literature) allele deletion. Five different sequence variations were identified by comparing the *HvGA20ox2* gene sequence of Deba Abed with the tall barley cultivars (AC Metcalfe, Hamelin and Valticky). The deletion of a single “A” and a “GTTA” insertion were located in the untranslated region of exon 1 in positions 42 and 64, respectively. The 4-bp insertion in the *sdw1.c* allele was further confirmed by using barley varieties with known genotype (Fig. 3). In addition, two synonymous mutations were also detected at positions 659 (coding sequence of exon 1, G/A transition) and 3161 (coding sequence of exon 3, C/G transversion). An A/C transversion was also detected at position 3321 in the 3’ UTR region (Fig. 1e). However, none of the synonymous mutations in coding region and the transversion in 3’ UTR is expected to explain the dwarf phenotype.

In contrast to *sdw1.c* and *sdw1.d* alleles, all primer combinations of the whole gene in Additional file 2: Table S2 failed to amplify any fragment from the *sdw1.a* mutants. PCR amplification analyses spanning the *HvGA20ox2* gene locus and the neighboring genes identified a possible deletion of the whole *HvGA20ox2* gene in *sdw1.a* varieties (data not shown).

### Mapping the *HvGA20ox2* gene in the Baudin/AC Metcalfe population

Two molecular linkage maps have been constructed for the Baudin/AC Metcalfe DH (double haploid) population. The first map was constructed with 178 DH lines and 234 SSR and AFLP markers [18]. The second map has 12,998 SNP tags anchored to seven chromosomes, spanning a cumulative 967.6 cM genetic distance [19]. In both maps the 7-bp indel polymorphism mapped to the expected location on chromosome 3H (data not shown).

Plant heights from three different field trials were used for QTL analysis. The average height of *sdw1.d* allelic plants was 16 to 19 cm shorter than the wild type plants in all trials (Additional file 1: Figure S2). However, large variation in plant height was observed within an allelic class (Additional file 1: Figure S2). A major QTL was identified for plant height and explained 37.2–44.5% of the plant height variation (Additional file 2: Table S3). The QTL peak co-located with the *HvGA20ox2* gene-specific marker (Additional file 1: Figure S3).

### Association analysis of the gene-specific marker in a natural population

One hundred and ninety-seven barley varieties, breeding lines and landraces were collected from Australia, Africa, China, European, North and South America and their plant heights varied from 50 to 105 cm. Of those, 28 accessions had the 7-bp deletion, three accessions had the 4-bp insertion while two did not yield an amplification product (Table 1). The 7-bp deletion points to the *sdw1.d* allele, the 4-bp insertion points to the *sdw1.c* allele and the lack of amplification points to the *sdw1.a* allele. Twenty-one barley accessions with the *sdw1.d* allele belong to the obvious dwarf types, with heights varying from 50 to 70 cm. Seven lines with the *sdw1.d* allele have a medium stature, from 75 to 80 cm. One *sdw1.c* allelic barley variety Tx9425 is the dwarf type. The two *sdw1.a* allelic barley varieties Yerong and Yan90260 are of the dwarf type. The *sdw1.a* and *sdw1.d* alleles explained 29% of plant height variation in the 197 barley varieties (P < 0.0001). We only detected the *sdw1.a* and *sdw1.d* alleles in modern barley varieties. The results provide further support for GA20 oxidase 2 (*HvGA20ox2*) as the functional gene for the *sdw1* locus. We also observed that 52 barley varieties/lines displayed the short stature without the *sdw1.a*, *sdw1.c* and *sdw1.d* alleles in this population.

**Table 1:** Barley varieties used in this study, their origins, plant height (Ht) and their genotype at the *sdw1* gene locus

No	Variety - Association	ORIG	Ht (cm)	Genotype^a^
1	Sahara	Africa	105	WT
2	Cevada de 2 Ordens	Australia	85	WT
3	Cevada de 6 Ordens	Australia	95	WT
4	Baudin	Australia	55	sdw1.d
5	Fitzgerald	Australia	70	WT
6	Gairdner	Australia	65	sdw1.d
7	Hamelin	Australia	75	WT
8	Stirling	Australia	85	WT
9	Vlamingh	Australia	75	WT
10	Bass	Australia	60	sdw1.d
11	WABAR2252	Australia	75	WT
12	Yambla	Australia	75	WT
13	Brindabella	Australia	53	WT
14	TF026	Australia	65	WT
15	YF374	Australia	65	WT
16	Tx9425	Australia	70	Sdw1.c
17	Yerong	Australia	62	sdw1.a
18	WB229	Australia	75	WT
19	Hindmarsh	Australia	70	WT
20	Mundah	Australia	75	WT
21	Macquarie	Australia	65	WT
22	Barque 73	Australia	87.5	WT
23	Clipper	Australia	77.5	WT
24	Flagship	Australia	80	WT
25	Schooner	Australia	80	WT
26	Skiff	Australia	60	WT
27	Commander	Australia	75	WT
28	WI 4262	Australia	70	sdw1.d
29	VB0432-B2	Australia	60	sdw1.d
30	WA12428	Australia	75	WT
31	WA13255	Australia	70	WT
32	WA13581	Australia	75	WT
33	WA13582	Australia	80	WT
34	WA13583	Australia	80	WT
35	WA13585	Australia	70	WT
36	WA13586	Australia	80	WT
37	WA13588	Australia	80	WT
38	WA13589	Australia	75	WT
39	WA13590	Australia	75	WT
40	WA13591	Australia	70	WT
41	WA13597	Australia	80	WT
42	WA13602	Australia	60	WT
43	WA13603	Australia	65	WT
44	WA13604	Australia	85	WT
45	EB1110	Australia	80	WT
46	EB1111	Australia	65	WT
47	EB1112	Australia	75	WT
48	NBX05019-08-099	Australia	66	WT
49	NBX05020-08-057	Australia	70	WT
50	WA13619	Australia	75	WT
51	WA11645	Australia	65	WT
52	Fleet	Australia	75	WT
53	Keel	Australia	72	WT
54	WA12423	Australia	80	WT
55	WA13233	Australia	75	WT
56	WA12438	Australia	80	WT
57	WA13237	Australia	85	WT
58	WA13240	Australia	75	WT
59	WA13241	Australia	75	WT
60	WA13242	Australia	65	WT
61	WA13245	Australia	85	WT
62	WA13251	Australia	65	WT
63	WA13261	Australia	78	WT
64	Buloke	Australia	87	WT
65	Br2	Brazil	75	WT
66	TR06106	Canada	60	WT
67	SB03180	Canada	65	WT
68	HB705	Canada	70	WT
69	BM9919-90	Canada	85	WT
70	H95027004	Canada	80	sdw1.d
71	H95032005	Canada	70	WT
72	H96009015001	Canada	80	WT
73	H96009015002	Canada	80	WT
74	M94060003	Canada	80	WT
75	H95030001	Canada	75	WT
76	H95039003	Canada	80	WT
77	H95042004	Canada	75	WT
78	H95052002	Canada	70	WT
79	M94257001	Canada	90	WT
80	H95011020	Canada	75	WT
81	H95011024	Canada	70	WT
82	H95056002	Canada	85	WT
83	H95056005	Canada	70	WT
84	YHZWB	China	95	WT
85	B1052	China	65	WT
86	B1067	China	55	WT
87	B1079	China	80	WT
88	B1064	China	95	WT
89	B1133	China	90	WT
90	B1043	China	70	WT
91	B1118	China	65	WT
92	B1100	China	100	WT
93	B1121	China	80	WT
94	JSELM	China	90	WT
95	PTWDDM 2	China	85	WT
96	PTWDDM 3	China	86	WT
97	PTWDDM 4	China	87	WT
98	PTWDDM 5	China	90	WT
99	PTWDDM 6	China	88	WT
100	PTWDDM 8	China	80	WT
101	93-3143	China	80	WT
102	Aizao 3	China	75	WT
103	CxHKSL	China	90	Sdw1.c
104	DYSYH	China	90	WT
105	Hu93-043	China	65	WT
106	Lixi 143	China	75	WT
107	RGZLL	China	85	WT
108	Xiaojiang	China	80	WT
109	YUQS	China	70	WT
110	YWHKSL	China	105	WT
111	YYXT	China	65	WT
112	Zhepi 2	China	60	WT
113	ZUG293	China	70	WT
114	ZUG403	China	75	WT
115	Yan89110	China	90	WT
116	Yan90260	China	65	sdw1.a
117	Yiwu Erleng	China	70	WT
118	YPSLDM	China	100	WT
110	YSMI	China	80	WT
121	YSM3	China	75	WT
122	YU6472	China	65	WT
123	W2	China	80	WT
124	W1	China	76.8	WT
125	KM 123	Czech Republic	55	WT
126	Pavlovicky	Czech Republic	100	WT
127	K 70	Czech Republic	95	WT
128	Czech Landrace-243	Czech Republic	70	WT
129	IEDNVT 1	EU	75	sdw1.d
130	IEDNVT 2	EU	80	sdw1.d
131	IEDNVT 3	EU	75	sdw1.d
132	IEDNVT 4	EU	80	sdw1.d
133	INEDNVT 5	EU	75	sdw1.d
134	INEDNVT 6	EU	80	sdw1.d
135	Adagio	France	60	sdw1.d
136	Naso nijo	Japan	80	WT
137	Noire Maroc	Morocco	80	WT
138	Precoce du Maroc	Morocco	75	WT
139	Barlis	Morocco	100	WT
140	Moroccan Landrace	Morocco	85	WT
141	Portuguese landrace	Portugal	75	WT
142	Boa Fe	Portugal	85	WT
143	cevada Preta	Portugal	95	WT
144	CSK-81-556	Slovakia	75	WT
145	WVA 18	South Africa	60	WT
146	WVA 19	South Africa	85	WT
147	WVA 20	South Africa	65	sdw1.d
148	WVA 22	South Africa	50	sdw1.d
149	WVA 24	South Africa	70	WT
150	WVB 7	South Africa	60	sdw1.d
151	WVB 9	South Africa	70	sdw1.d
152	WVB 22	South Africa	50	sdw1.d
153	WVB 29	South Africa	60	sdw1.d
154	WVB 33	South Africa	60	sdw1.d
155	WVB 34	South Africa	50	sdw1.d
156	WVB 35	South Africa	55	sdw1.d
157	WVC 3	South Africa	60	sdw1.d
158	HOR13461	Spain	70	WT
159	Spanish Landrace-333c	Spain	105	WT
160	Spanish landrace 355	Spain	85	WT
161	Spanish landrace 336d	Spain	80	WT
162	Spanish landrace 352	Spain	75	WT
163	Spanish landrace 349b	Spain	105	WT
164	Spanish landrace 349	Spain	105	WT
165	Spanish landrace 316	Spain	70	WT
166	Spanish landrace 338c	Spain	90	WT
167	Spanish landrace 333	Spain	95	WT
168	Spanish landrace 309d	Spain	80	WT
169	HOR12517	Spain	72.5	WT
170	Keka	Spain	85	WT
171	Rosa	Spain	100	WT
172	HOR 13461	Spain	90	WT
173	NFC Tipple	UK	55	sdw1.d
174	Waggon	UK	65	WT
175	Cocktail	UK	65	sdw1.d
176	Wicket	UK	60	sdw1.d
177	Flagon	UK	75	WT
178	Braemar	UK	65	sdw1.d
179	2B03-3604	USA	70	WT
180	2B03-3631	USA	75	WT
181	2B03-3785	USA	55	WT
182	2B03-3830	USA	75	WT
183	2B03-3859	USA	65	WT
184	2B03-3882	USA	80	WT
185	Z034P013Q	USA	80	WT
186	Z034P116Q	USA	60	sdw1.d
187	Z035R014S	USA	80	WT
188	Z051R077S	USA	70	WT
189	Z051R101S	USA	65	WT
190	Z052R091S	USA	80	WT
191	Z055O012O	USA	65	WT
192	Z090M066M	USA	65	WT
193	Z118M006M	USA	80	WT
194	Dayton	USA	75	Sdw1.c
195	Numar	USA	75	WT
196	MAR-86-E1138		90	WT
197	MAR-82-E1138		80	WT

^a^ WT: wild type; sdw1.d: *sdw1.d* allele; sdw1.a: *sdw1.a* allele; sdw1.c: *sdw1.c* allele

### Transcription levels of genes encoding the final steps of GA biosynthesis

**Fig. 4:**
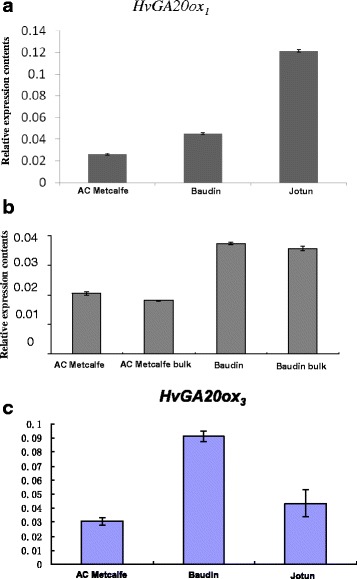
Relative gene expression levels of *HvGA20ox1*and *HvGA20ox3*. **a**: transcription level of *HvGA20ox1* at stem elongation stage in AC Metcalfe (wild type), Baudin (*sdw1.d* allele) and Joutn (*sdw1.a* allele); **b**: bulk-segregating analysis of *HvGA20ox1* gene expression at tillering stage in Baudin/AC Metcalfe DH population, each bulk contained 20 DH lines with different alleles of the *HvGA20ox2* gene; **c**: transcription level of *HvGA20ox3* at stem elongation stage in AC Metcalfe (wild type), Baudin (*sdw1.d* allele) and Joutn (*sdw1.a* allele)

Our previous result demonstrated that the mutations in *sdw1.d* and *sdw1.a* reduced the gene expression of *HvGA20ox2* [20]. In this study, we also measured the expression of the other two homologous genes *HvGA20ox1* and *HvGA20ox3* (Fig. 4a,c). It is surprised that the expression level of *HvGA20ox1* was 1.7 times higher in Baudin (*sdw1.d*) and 4.7 times higher in Jotun (*sdw1.a*) while *HvGA20ox3* showed three times higher in Baudin and 1.4 times higher in Jotun. The result suggests that partial or total loss of function of *HvGA20ox2* can be compensated by other GA20 oxidases, especially *HvGA20ox1*.

To further confirm if the increased expression of *HvGA20ox1* was due to partial loss of function of *HvGA20ox2*, we conducted a bulked segregant analysis of gene expression in the Baudin (*sdw1.d*)/AC Metcalfe (tall) DH population. The expression level of the *sdw1.d* bulk matched with the *sdw1.d* parent Baudin, with higher expression and reversed trend observed in the tall bulk and AC Metcalfe (tall parent) (Fig 4b). From those results we conclude that partial loss (*sdw1.d*) or total loss (*sdw1.a*) of *HvGA20ox2* may be compensated by increased expression of *HvGA20ox1*.

## Discussion

Modification of the gibberellin biosynthetic and signal transduction pathways was a crucial step in crop breeding, as it conferred the agronomically important semi-dwarf phenotype [21]. The rice green revolution gene *sd1* was the result of reduced function of GA 20-oxidase-2 [3]. The GA 20-oxidases are involved in the later steps of GA biosynthesis, in which GA_53_ is converted into GA_44_ [17]. It is now clear that reduced function of the GA 20-oxidase gene leads to reduction in plant height in rice. A previous study has demonstrated that the *sdw1* gene may be orthologous to the rice *sd1* gene [13]. However, it is not clear how the gene structure changes resulted in dfiierent functional alleles. In this study, we characterized a full-length copy and alleles of the barley *HvGA20ox2* gene, which has a conserved gene structure when compared to the rice *sd1* gene. Sequence similarity analysis showed that the predicted protein of the barley *HvGA20ox2* gene shared 83.1% of identity to its rice ortholog.

Four alleles have been reported at the *sdw1* locus. In this study, we characterized the *HvGA20ox2* gene from three independent mutants. The *sdw1.a* allele might be the result of a total deletion of the *HvGA20ox2* gene. Nearly no expression of *HvGA20ox2* was detected for the *sdw1.a* mutant (Jotun) previously [20], which was consistent with a total deletion of the *HvGA20ox2* gene, as our study suggests. A recent study demonstrated that *sdw1.e* (mutant line ‘Ris∅ no. 9265’) also resulted from a total deletion of the *HvGA20ox2* [22]. The *sdw1.c* allele has a 1-bp deletion and a 4-bp “GTTA” insertion in the untranslated region of exon1, respectively. The *sdw1.d* (Diamant) allele is caused by a 7-bp deletion in exon1, which resulted in coding frame shifts and premature translation termination. As there is an internal ATG, the *sdw1.d* (Diamant) allele may lead to a truncated protein with a conserved domain of the 2-oxoglutarate (2OG) and Fe(II)-dependent oxygenase superfamily. Thus, the *sdw1.d* (Diamant) allele still maintains partial function of GA 20-oxidase. Sequencing of different alleles at the *sdw1* locus points to *HvGA20ox2* as the functional gene responsible for the phenotype.

Based on our sequencing results, we designed an allele-specific marker. As expected, the allele-specific marker co-segregated with a major QTL controlling plant height in the DH population of Baudin/AC Metcalfe. The gene-specific marker was further tested in a natural population. We found the *sdw1.a* and *sdw1.d* alleles only in modern barley varieties and associated with plant height. These results provide further support for *HvGA20ox2* as the functional gene of the *sdw1* locus. However, the molecular marker for the 4 bp insertion in the *sdw1.c* allele seems not associated with plant height in the natural population. We speculate that the 1 bp deletion may be more important for the gene function in the *sdw1.c* allele as the sdw1.d allele.

Until now, no malting barley variety has been developed from the *sdw1.a* allele. Bioactive gibberellins are not only essential regulators for barley growth and development, but are also essential for malting and brewing [23]. It is expected that the deletion of the *HvGA20ox2* gene in *sdw1.a* allele would result in reduced GA biosynthesis during the malting process. This would explain why the *sdw1.a* allele has been used exclusively in feed barley.

A recent study in *Arabidopsis thaliana* reported 21 independent loss-of-function alleles at GA locus 5 (GA5), which encodes gibberellin 20-oxidase 1 (*GA20ox1*), causing semi-dwarfness [24]. These results suggest that GA 20-oxidase might be a hot spot for phenotypic variation in crop and other plant species. Further research is required to establish whether there is further allelic variation in *HvGA20ox2* in barley.

The predicted protein of the barley *HvGA20ox2* gene shared high identity with the *Aegilops* and wheat orthologs (Fig. 2), which raises the question why no such semi-dwarf mutants have been identified in these species thus far. Such mutants have already demonstrated great potential to increase yield in rice and barley, and thus it seems worthwhile creating similar mutants in wheat as an alternative source of dwarfing genes. Our results further demonstrate that GA20 oxidase homologs can functionally compensate for each other (Fig. 4b). This means that to achieve a similar feat in wheat, GA20 oxidase expression in all three genomes would have to be modified simultaneously. Advances in sequencing and gene editing technologies may provide an efficient approach to identifying or producing such mutants in wheat.

Previously, a SNP in intron 2 was detected between semi-dwarf barley variety Baudin and tall variety AC Metcalfe [13]. The SNP marker was mapped to chromosome 3H in the double haploid population of Baudin/AC Metcalfe, while co-segregating with plant height [13]. However, this SNP is not unique for the *sdw1.d* allele. In contrast, the allele-specific marker in this study can be used as a diagnostic test for the *sdw1.a*, *sdw1.d* and wild-type alleles.

The *sdw1* alleles explained part of the height variation in both the DH population and the test barley varieties. Some barley varieties without the *sdw1.a* and *sdw1.d* alleles also displayed short stature. These results indicated that some novel dwarfing genes have already used to breed barley varieties [6, 9, 25–29]. We also observed the plant height variation within allele classes was much greater than the variation between *sdw1.d* allele class and wild type class. This indicated that some novel dwarfing genes also responsible for the height variation between Baudin and AC Metcalfe [6, 9, 25–29].

## Methods

### Genetic materials and agronomic traits

The medium tall barley varieties used in this study included AC Metcalfe, Valticky (parent of Diamant), and Hamelin. The semi-dwarf barley varieties Diamant and Baudin represent the *sdw1.d* allele. The *sdw1.d* allele in Baudin was from Triumph, which derived its *sdw1.d* gene from Diamant. The barley variety Deba Abed represents the *sdw1.c* (*denso*) allele. Jotun is the *sdw1.a* mutant. Yerong is a semi-dwarfing dual-purpose (feed and graze) barley variety carrying *sdw1.a* gene [30].

A doubled haploid population comprising 178 lines was generated via anther culture from the F1 progeny of a Baudin/AC Metcalfe cross. The 197 barley varieties and lines used in this study were collected from Australia, Africa, Europe, North and South America, and are listed in Table 1.

The mapping population (178 DH lines) with its parents and the 197 barley accessions were planted at three sites in Western Australia. The field trial sites were located in the high rainfall agricultural zone, in order to achieve the maximum growing potential for the semi-dwarf genotypes. The DH lines and parents were planted in 1 × 5 m plots and the same randomized design was used at each site for convenience. Parental and local barley varieties were used as grid controls for spatial analysis.

### Cloning of *HvGA20ox2* gene from barley varieties

Polymerase chain reaction (PCR) primers were designed from the cloned fragments of the *HvGA20ox2* gene [13] and barley genome sequencing information (Additional file 2: Table S2). The relative positions of each primer to the *HvGA20ox2* gene are shown in Additional file 1: Figure S1. All primers were synthesized by Gene Works Pty. Ltd. (Australia). The PCR reactions consisted of 50 ng genomic DNA as template, 0.1 μM of each primer, in a final volume of 10 μl containing 1 × PCR buffer, 1.5 mM MgCl_2_, 0.2 mM dNTP, and 0.5 U Taq polymerase (Bioline, Australia). The PCR reactions were performed using the following program: denaturation at 94 °C for 3 min, followed by 35 cycles of 94 °C for 30 s, annealing for 45 s and extension at 72 °C for 1 min, and a final extension at 72 °C for 5 min. The optimal annealing temperature of each pair of primer combination was determined by gradient PCR. The PCR products were cloned into pGEM-T Easy Vector (Promega), and at least two independent clones from each PCR product were sequenced using an automated sequencing system (ABI 377, Applied Biosystems).

### Sequence assembly and alignment

The target sequences of each variety were assembled by the SeqMan program (DNAStar). Clustal X2 was used for multiple sequence alignment. The exon and intron, and protein sequences of the *HvGA20ox2* gene from each variety were identified by using BLASTN, TBLASTN, and online gene prediction software FGENESH (http://linux1.softberry.com/berry.phtml?topic=fgenesh&group=programs&subgroup=gfind). The orthologs of the barley *HvGA20x2* gene from other grass species and *Arabidopsis* were confirmed by BLASTP. The identity of the deduced amino acid of the *HvGA20x2* gene among the orthologs was analyzed by DNAStar. Phylogenetic trees of the predicted proteins of the barley *HvGA20ox2* gene, including the orthologous proteins *HvGA20ox1* and *HvGA20ox3* was constructed using MEGA 6.0 by maximum likelihood approach, and the confidence of the nodes was evaluated using 1000 bootstrap replications.

### Real-time quantitative RT-PCR

RNA was extracted from the stems at tillering or stem elongation stage using a Spin Column Plant total RNA Purification Kit(Sanggon Biotech (Shanghai) Co., Ltd. cDNA was prepared from 1 μg RNA using AMV First Strand cDNA Synthesis Kit(Sanggon Biotech (Shanghai) Co., Ltd). qPCR reactions were performed using SYBR Green (SG Fast qPCR Master Mix(High Rox), BBI) and the Applied Biosystems Stepone plus Real-time PCR System. The Real-time PCR assays were performed in triplicate for each cDNA sample. To determine transcription levels of barley *HvGA20ox2* and genes encoding the final steps of GA biosynthesis, *HvACTIN* and *HvGAPDH* were employed as reference genes for barley. The oligonuleotide sequences used for quantitative RT-PCR are listed in Additional file 2: Table S4.

To determine if other genes are regulated by *HvGA20ox2*, 20 doubled haploid lines from the Baudin/AC Metcalfe population were selected based on the genotype of the *HvGA20ox2* gene to construct two pools (*sdw1.d* and wild type) for measurement of the expression of other genes in the GA biosynthesis pathway. Three biological repeats were used for RNA extraction.

### Verification of the denso allele in a DH population

Presence of the *sdw1.d* allele was verified in the DH population of Baudin/AC Metcalfe and barley cultivars. Genomic DNA was extracted from young leaves using the standard CTAB protocol. DNA samples were quantified using the Nanodrop equipment and adjusted to a final concentration of 50 ng/μL for PCR. Primers used are listed in Additional file 2: Table S1. PCR amplification conditions were as described above. The PCR products were separated in 6% PAGE gels.

### QTL analysis for plant height

The software package MapQTL 5.0 was used to conduct QTL analysis for plant height after import of the files for genotypes, phenotypes and genetic maps. Interval analysis was first performed to estimate the closest markers associated with plant height, followed by multiple QTL model (MQM) analysis. LOD threshold values applied to declare the presence of a QTL were estimated by performing whole-genome wide permutation tests using 10,000 permutations. The QTL map was then generated using Mapchart 2.2.

## Conclusions

Our research provided further evidence that the gibberellin 20-oxidase gene (*HvGA20ox2*) is the functional gene for the barley *sdw1* mutants. The *sdw1.d* allele from Diamant is due to a 7-bp deletion in exon 1, while the *sdw1.c* allele from Abed Denso has 1-bp deletion and a 4-bp insertion in the 5’ untranslated region. The *sdw1.a* allele from Jotun resulted from a total deletion of the *HvGA20ox2* gene. Partial or total loss of function of the *HvGA20ox2* gene could be compensated by enhanced expression of its homolog *HvGA20ox1* and *HvGA20ox3*. A diagnostic molecular marker was developed to differentiate between the wild-type, *sdw1.d* and *sdw1.a* alleles and another molecular marker for differentiation of *sdw1.c* and *sdw1.a.* Further research is required to establish whether the truncated protein could maintain partial function and whether there is further allelic variation in* HvGA20ox2* in barley.

## Additional files

Additional file 1: Figure S1. Structure of barley *HvGA20ox2* gene and the relative position of the primers used in this study. **Figure S2**. Plant height (cm) variation in Baudin/AC Metcalfe DH population from three independent field trials (SP-Ht: South Perth plant height; KD Ht: Plant height in Kendup trials. **Figure S3**. A major QTL for plant height co-segregated with *HvGA20ox2* on chromosome 3H. The genetic map is based on Zhou et al. (2015). (ZIP 118 kb)
Additional file 2: Table S1. Identity of the deduced amino acid sequence of the *HvGA20ox2* gene with selected orthologs. **Table S2**. Primers used to amplify the *HvGA20ox2* gene and inspect *sdw1* allelic variations. **Table S3**. Barley varieties used in this study and their genotype at the *sdw1* gene locus. **Table S4**. The oligonuleotide sequences used for quantitative RT-PCR for different genes. (DOCX 28 kb)

## Abbreviations

AFLP: Amplified restriction fragment polymorphism
cM: Centimorgan
DH: Double haploid
GA: Gibberellic acid
PCR: Polymerase chain reaction
QTL: Quantitative trait loci
*Rht*: Reduced height
*sd1*: Semidwarf-1
*sdw1*: Semi-dwarf 1
SNP: Single nucleotide polymorphism
SSR: Simple sequence repeats
