# Supplementary figures and images for: Characterization of the sdw1 semi-dwarf gene in barley
Source: BMC Plant Biol. 2017 Jan 13;17:11. doi: 10.1186/s12870-016-0964-4 (PMC5237212; doi:10.1186/s12870-016-0964-4)

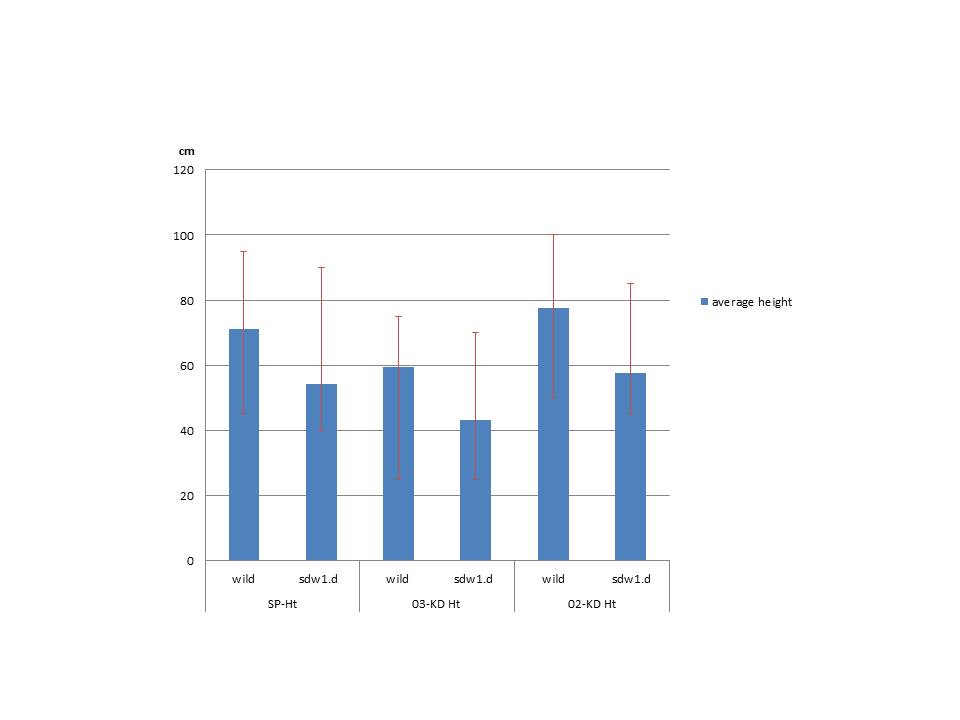

Supplement: Additional file 1: Figure S1. — Structure of barley HvGA20ox2 gene and the relative position of the primers used in this study. Figure S2. Plant height (cm) variation in Baudin/AC Metcalfe DH population from three independent field trials (SP-Ht: South Perth plant height; KD Ht: Plant height in Kendup trials. Figure S3. A major QTL for plant height co-segregated with HvGA20ox2 on chromosome 3H. The genetic map is based on Zhou et al. (2015). (ZIP 118 kb) [file 12870_2016_964_MOESM1_ESM.zip › Fig S2.jpg]

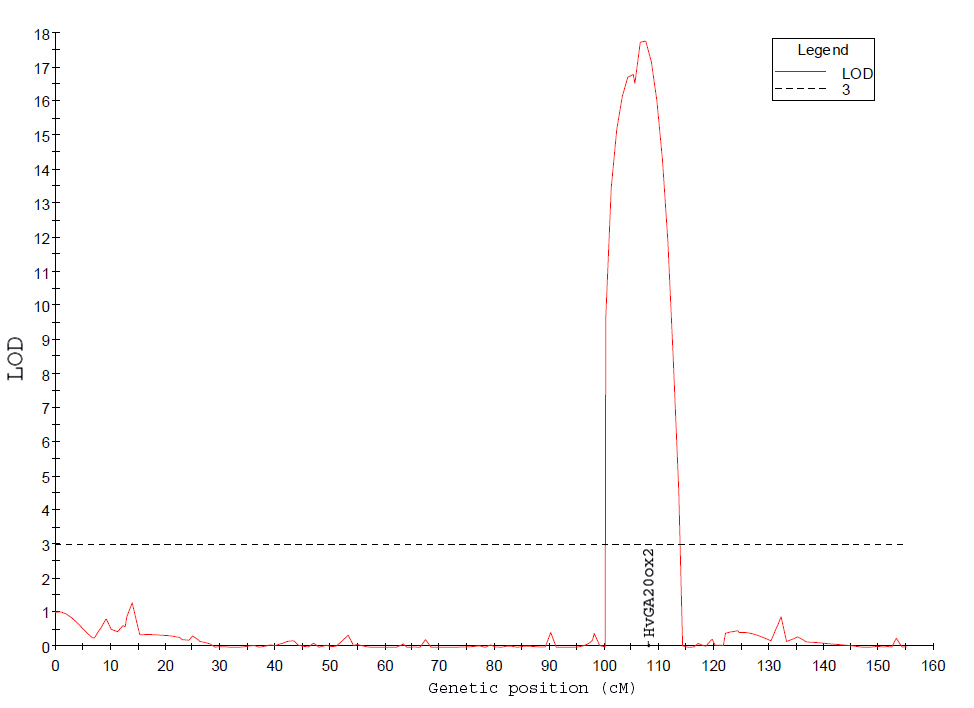

Supplement: Additional file 1: Figure S1. — Structure of barley HvGA20ox2 gene and the relative position of the primers used in this study. Figure S2. Plant height (cm) variation in Baudin/AC Metcalfe DH population from three independent field trials (SP-Ht: South Perth plant height; KD Ht: Plant height in Kendup trials. Figure S3. A major QTL for plant height co-segregated with HvGA20ox2 on chromosome 3H. The genetic map is based on Zhou et al. (2015). (ZIP 118 kb) [file 12870_2016_964_MOESM1_ESM.zip › Fig S3.jpg]

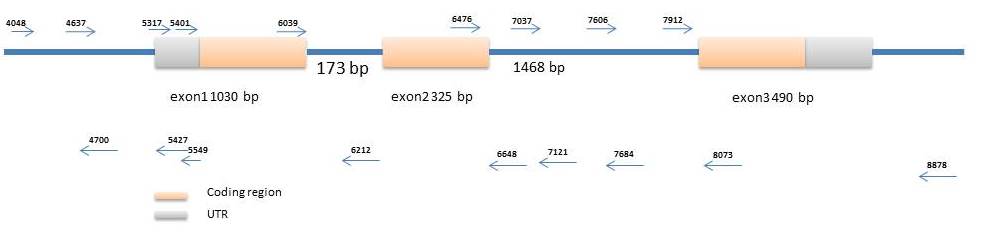

Supplement: Additional file 1: Figure S1. — Structure of barley HvGA20ox2 gene and the relative position of the primers used in this study. Figure S2. Plant height (cm) variation in Baudin/AC Metcalfe DH population from three independent field trials (SP-Ht: South Perth plant height; KD Ht: Plant height in Kendup trials. Figure S3. A major QTL for plant height co-segregated with HvGA20ox2 on chromosome 3H. The genetic map is based on Zhou et al. (2015). (ZIP 118 kb) [file 12870_2016_964_MOESM1_ESM.zip › Fig S1.jpg]
