# Supplementary material for: Characterization of the sdw1 semi-dwarf gene in barley
Source: BMC Plant Biol. 2017 Jan 13;17:11. doi: 10.1186/s12870-016-0964-4 (PMC5237212; doi:10.1186/s12870-016-0964-4)
Supplement: Additional file 2: Table S1. — Identity of the deduced amino acid sequence of the HvGA20ox2 gene with selected orthologs. Table S2. Primers used to amplify the HvGA20ox2 gene and inspect sdw1 allelic variations. Table S3. Barley varieties used in this study and their genotype at the sdw1 gene locus. Table S4. The oligonuleotide sequences used for quantitative RT-PCR for different genes. (DOCX 28 kb) [file 12870_2016_964_MOESM2_ESM.docx]

Table S1 Identity of the deduced amino acid sequence of the *HvGA20ox2* gene with selected orthologs

| Proteins | No. | 1 | 2 | 3 | 4 | 5 | 6 | 7 | 8 | 9 | 10 | 11 | 12 |
| --- | --- | --- | --- | --- | --- | --- | --- | --- | --- | --- | --- | --- | --- |
| AAL87949 rice | 1 | — | 51.1 | 46.8 | 77.1 | 77.9 | 76.9 | 76.9 | 48.0 | 81.5 | 76.1 | 79.7 | 79.4 |
| AAT49058 barley GA 20-oxidase 1 | 2 |  | — | 56.9 | 50.6 | 49.7 | 49.7 | 50.6 | 57.8 | 51.4 | 51.1 | 52.2 | 51.1 |
| AAT49059 barley GA 20-oxidase 3 | 3 |  |  | — | 48.5 | 48.7 | 49.0 | 48.5 | 49.6 | 49.0 | 45.4 | 48.7 | 48.7 |
| Barley HvGA20ox2 | 4 |  |  |  | — | 94.0 | 95.4 | 95.9 | 46.9 | 78.0 | 74.7 | 75.8 | 74.4 |
| CDM85079 wheat | 5 |  |  |  |  | — | 95.2 | 90.4 | 47.7 | 76.5 | 77.1 | 73.6 | 72.9 |
| EMT17460 *Aegilops tauschii* | 6 |  |  |  |  |  | — | 91.6 | 48.8 | 78.2 | 75.9 | 74.5 | 74.3 |
| HvGA20ox2 *sdw1.d* allele | 7 |  |  |  |  |  |  | — | 46.9 | 79.2 | 77.5 | 77.0 | 75.9 |
| NP_194272 *Arabidopsis thaliana* | 8 |  |  |  |  |  |  |  | — | 49.1 | 49.3 | 49.9 | 48.0 |
| XP_002456751 *Sorghum bicolor* | 9 |  |  |  |  |  |  |  |  | — | 74.5 | 85.5 | 88.9 |
| XP_003567337 *Brachypodium* spp | 10 |  |  |  |  |  |  |  |  |  | — | 73.0 | 71.0 |
| XP_004970813 *Setaria italica* | 11 |  |  |  |  |  |  |  |  |  |  | — | 84.7 |
| XP_008654721 *Zea Mays* | 12 |  |  |  |  |  |  |  |  |  |  |  | — |

Table S2 Primers used to amplify the *HvGA20ox2* gene and inspect *sdw1* allelic variations

| Amplification position | Forward primer | Sequence (5'-3') | Reverse primers | Sequence (5'-3') | Expected size [bp] |
| --- | --- | --- | --- | --- | --- |
| 4048-4700 | sdw4048F | TAAGTCTCCTTCAGAGTTCACCT | sdw4700R | CAATTCCCATATCAGGTGTAT | 652 |
| 4637-5427 | sdw4637F | TCTGGAGAAAGGAGACTAAAT | sdw5427R | GGTTCTTGCTGAGCGGTC | 790 |
| 5317-6212 | sdw5317F | CCCGATTACAAATACCCC | sdw6212R | GTGTTCTCTCCTCAGTACTCTG | 895 |
| 6039-6648 | sdw6039F | CGGACTACGAGCCAATGG | sdw6648R | AGATTTAGTAAGCGTAAGTAGGT | 610 |
| 6476-7121 | sdw6476F | AGGTCCTCGTCGACGGCGACT | sdw7121R | AGTTCCTTGAGGCATCGT | 645 |
| 7037-7684 | sdw7037F | CCGAAGTGACATAGCGAC | sdw7684R | CGAACAGACAAAGCGGAG | 647 |
| 7606-8073 | sdw7606F | TCGCCACGGCACTGTATC | sdw8073R | TTCACCACCGCCCGGTGCAG | 467 |
| 7912-8878 | sdw7912F | GACGGATACCGAAATGAAAACG | sdw8878R | CCGCATAAAAGACTCGGGACG | 966 |
| sdw1.d indels | sdw5401F | GGTGCTCCAGACCGCTCAG | sdw5549R | CGGCGGAGGGGTCAATG | 149/142 |
| Sdw1.c indels | MC40861P3F | TATGGCGTGACCAAAGGTTC | MC40861P4R | CACCAATCCACCACGAAGA | 242/245 |

Table S3. Phenotypic variation of plant height explained by *HvGA20ox2* gene specific molecular marker

(mu_A: average height of genotype Baudin; mu_B: average height of genotype AC Metcalfe; %Expl: QTL explained phenotypic variation)

| Trials | Group | Position | Locus | LOD | mu_A | mu_B | Variance | % Expl. | Additive |
| --- | --- | --- | --- | --- | --- | --- | --- | --- | --- |
| height | 3 | 103.6 | LG03_56965410 | 9.36 | 49.0648 | 70.099 | 185.887 | 37.2 | -10.5171 |
| 02-KDHt | 3 | 105.5 | LG10_116465883 | 11.61 | 58.3416 | 76.1459 | 98.7118 | 44.5 | -8.90218 |
| 03-KD Ht | 3 | 106.1 | LG03_98865694 | 10.36 | 43.4189 | 58.0535 | 74.1575 | 41.8 | -7.31732 |

Table S4 The oligonuleotide sequences used for quantitative RT-PCR for different genes

| Primer Name | Primer sequence |
| --- | --- |
| HvGA20ox2-F | CCATCATGCGGTGCAACTA |
| HvGA20ox2-R | AAGGTGTCGCCGATGTTGA |
| HvGA20ox1-F | GCAGGGAGTGAGAAATCGACC |
| HvGA20ox1-R | CCATCCATGCATCGCTACCAC |
| HvGA3ox2-F | TCCGTCTCCACCATTGCTCGCTAG |
| HvGA3ox2-R | CTTGCTCAGGTGCGACGGCGT |
| HvGA2ox3-F | GCAGGTGCTGACCAACG |
| HvGA2ox3-R | GGTGCAATCCTCTGTGTCAA |
| HvActin-F | TGTTCCCAGGTATCGCTGAC |
| HvActin-R | GCCAGACTCGTCGTACTCATC |
| HvGAPDH-F | AGGCTATCAAGGCTGCTTCC |
| HvGAPDH-R | ACCCCACTCGTTGTCATACC |
